# Evolution of Cooperation Driven by Reputation-Based Migration

**DOI:** 10.1371/journal.pone.0035776

**Published:** 2012-05-16

**Authors:** Rui Cong, Bin Wu, Yuanying Qiu, Long Wang

**Affiliations:** 1 MOE Key Laboratory of Electronic Equipment Structure Design, School of Mechano-Electronic Engineering, Xidian University, Xi' an, China; 2 Center for Systems and Control, State Key Laboratory for Turbulence and Complex Systems, College of Engineering, Peking University, Beijing, China; University of Maribor, Slovenia

## Abstract

How cooperation emerges and is stabilized has been a puzzling problem to biologists and sociologists since Darwin. One of the possible answers to this problem lies in the mobility patterns. These mobility patterns in previous works are either random-like or driven by payoff-related properties such as fitness, aspiration, or expectation. Here we address another force which drives us to move from place to place: reputation. To this end, we propose a reputation-based model to explore the effect of migration on cooperation in the contest of the prisoner's dilemma. In this model, individuals earn their reputation scores through previous cooperative behaviors. An individual tends to migrate to a new place if he has a neighborhood of low reputation. We show that cooperation is promoted for relatively large population density and not very large temptation to defect. A higher mobility sensitivity to reputation is always better for cooperation. A longer reputation memory favors cooperation, provided that the corresponding mobility sensitivity to reputation is strong enough. The microscopic perception of the effect of this mechanism is also given. Our results may shed some light on the role played by migration in the emergence and persistence of cooperation.

## Introduction

Cooperation has been a puzzling problem to biologists, sociologists and economists since Darwin [1]–[3], because it benefits the defectors at costs to cooperators. In particular, it is interesting to investigate how cooperative behavior emerges in our human societies. Evolutionary game theory [4], [5] is a powerful framework for understanding the emergence of cooperation. Up to now, a number of mechanisms [6] have been proposed to address this issue, including kin selection [7], group selection [8], [9], direct reciprocity [10], [11], indirect reciprocity [12]–[16], and network reciprocity [17]–[21]. Particularly, indirect reciprocity and network reciprocity have attracted intensive attention in the study of human cooperative behaviors, because our societies are well captured by networks, and that indirect reciprocity, which means “helpful ones will be helped by others”, is more powerful in shaping our cooperative behaviors in human communities than in other animals.

Mobility, as an important characteristic of individuals in social network [22] and also a way to realize the coevolution [23]–[27], has recently received considerable attention in the study of cooperation. The population structure is often captured by spatial lattices [28]–[31]. Apart from population structures, the effects of mobility on the evolution of cooperation vary with movement patterns as well. To the best of our knowledge, there have been two types of forces that drive the migration. One is “random driven”. In this context, each individual is allowed to migrate to a nearby place randomly with a uniform probability on lattices [32], [33] or to move in random directions on continuous plane [34]. Further, moving individuals can be distinguished [35], [36] and endowed with different migration probabilities. The criteria of discrimination could be their strategies (cooperators or defectors) [37], or their influences [38]. The other is “payoff driven”. In these situations, an individual finds an empty site within some region that promises the highest payoff and then jumps to it (also called “success-driven”) [39], [40], or an individual will leave his current place if the payoff obtained does not meet the aspiration [41]–[43] or expectation [44]. In our human societies, reputation, as the fuel for the engine of indirect reciprocity, plays an important role in shaping our migration decisions. Yet it is still unclear how this “fuel”-driven migration affects cooperation. Considering this, we propose a “reputation-based migration” model to address this issue.

Reputation is a kind of social information by which individuals' past behaviors can be assessed. It has greatly contributed to evolution of cooperation in games of indirect reciprocity. The reputation can be simply evaluated by “image score” [13] or other criterions [15], [45]. In spatial games, individuals are distributed on the nodes of the networks, and the edges represent who plays with whom. An individual interacts with his direct neighbors and acquires the reputations of them. In reality, people tend to interact with those with good reputations that potentially benefit themselves, but leave those with bad ones to avoid being exploited in the future. Besides, individuals often have the information of their neighbors' reputations with the rest unknown. Inspired by these, we propose a model where migration is driven by the reputation. Therein, individuals evaluate the environments by the reputation scores of neighbors and themselves. An individual has a larger probability to leave his current place and to migrate to a random nearby site if his reputation is high comparing to his neighbors, revealing the fact that individuals favor a preferable environment but repel a nasty one [46]–[50]. For the updating of reputation, we incorporate memory effect [51] to the image scoring. To address how the reputation-based migration affects the evolution of cooperation, we consider how the two intrinsic parameters in the model affects the cooperation level: the reputation decaying rate, which depicts a cumulative effect of previous actions on reputation, and the mobility sensitivity to reputation, which characterizes how sensitively reputation influences the decisions of migration.

## Methods

In our model, the prisoner's dilemma game (PDG) is adopted as the paradigm to study the evolution of cooperation. For a typical PDG, each of the two players either cooperates (C) or defects (D). They both receive a reward 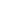 upon mutual cooperation and punishment 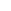 upon mutual defection. When confronted with a cooperator, the defector gains a temptation to defect 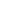, while the exploited cooperator acquires a sucker's payoff 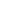. The ranking of the four payoff values is 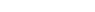. By setting 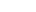, 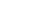, and 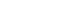, A simplified version of PDG [17] is obtained such that the game is controlled by a single parameter 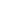 (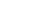), where 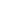 indicates the temptation to defect. For the network of social contacts, we assume that players are located on a square lattice of 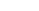 sites with periodic boundary conditions. Each site can be either empty or occupied by one individual. Empty sites represent spatial regions individuals can migrate to. We denote by 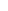 the density of population, defined as the ratio of population size to the number of all sites.

Initially, an equal percentage (50%) of cooperators (Cs) and defectors (Ds) are randomly distributed in the population. Individuals play PDG with their direct neighbors, and are updated asynchronously in a random sequential order. In each round, an individual is randomly picked up for updating. One round consists of two successive processes: *migration* and *imitation*. During the migration process, a randomly chosen individual 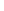 explores all the empty sites within the Moore neighborhood with distance 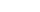 (the 8 neighboring sites). With probability 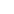, which is governed by the reputations of neighbors and himself, 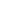 migrates to a random place among those empty sites. For the sake that 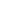 has neighbors to interact with, those empty sites who have neighbors (non-empty neighboring sites) will preferentially be chosen from at random. If there is no empty site within 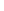
's neighborhood, 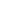 just stays where he is placed. In the imitation stage, 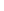 interacts with all his neighbors (von Neumann neighborhood). The payoffs are accumulated. Meanwhile, payoffs of 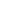
's neighbors are calculated in the same way. Then 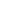 imitates the strategy of the neighbor who has the highest payoff (including himself), i.e., following a “best-take-over” rule. The reputation of 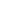 is updated at the same time. If 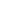 has no neighbor to interact with in his current place, both his strategy and his reputation remain unchanged during this round. A time step is defined as many rounds of games such that each individual will on average be chosen once. Therefore, the real number of rounds played in one time step varies with population density 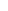. A key quantity that specifies the behavior of the system is the normalized fraction of cooperators 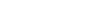 attained by averaging over many steps after the stationary phase is achieved, where 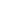 is the number of cooperators and 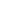 of all sites.

In order to account for the reputation effect in mobility, we define an individual 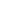
's reputation at time 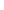 as
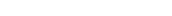
where 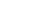 is 1 if 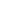 cooperates at time 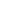, otherwise being 0, and 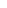 denotes the decaying rate. Thus an individual's reputation value is the weighted sum of the times he cooperated in the past games [52]. For 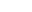, the memory effect vanishes and the reputation relies mainly on his current action; for 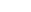, reputation value is accumulated since the outset of the evolution.

By comparing his reputation with those of his neighbors, 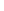 evaluates the environment and tends to leave when the environment is not so good. Thus the influence of reputation on mobility is implemented through determining the probability of an individual 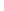 to leave his current site, namely
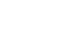
where 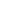 represents 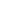
's neighbors and himself. The parameter 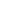 specifies an individual's mobility sensitivity to reputation. For 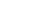, movement is insensitive to reputation and 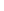 leaves with a probability equal to the inverse of the number of elements in set 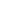, while 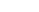 leads to the deterministic rule where 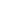 leaves with certainty if he is the one with the highest reputation among 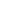, but stays still otherwise. For the case that 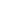 is isolated, we make a compulsory move by setting 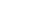. Considering that initially each individual has a reputation of zero, and thus no reputation records are available for guiding individuals' migration activities, we set 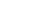 if all the members in 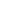 have a reputation equal to zero (and setting 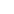 to be other constants in this case does not change the results qualitatively). Unlike models in ref [39], [40], where migration relies on information on strategies of all individuals in a large area, migration here requires local information only. Note that in our model, reputation takes effect in the migration process only, and thus no influence of reputation on strategy updating is involved.

**Figure 1:**
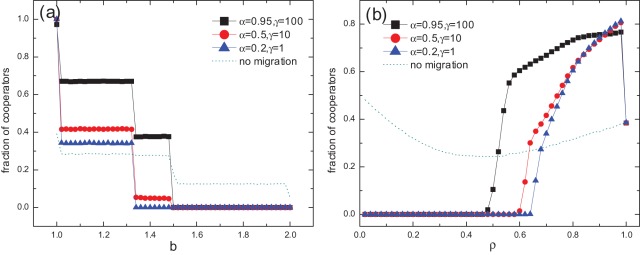
Effects of temptation to defect and population density on cooperation. (a) Fraction of cooperators 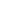 as a function of temptation to defect 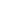 for population density 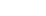 and (b) fraction of cooperators 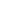 as a function of population density 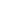 for temptation to defect 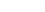, for different combinations of 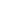 and 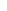. The cases without migration are plotted in dotted lines for comparison. Simulations are performed on a lattice of size 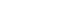. The quantity 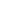 is obtained by averaging over 2000 time steps after 18000 time steps. Each data point results from an average over 100 independent realizations. We have checked that after 18000 time steps, the value of 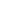 is stabilized.

## Results

In what follows, we present the results of numerical simulations. We first consider the influence of temptation to defect 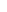 on the fraction of cooperators 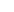. In the presence of reputation-based migration, 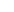 is promoted for low temptation to defect (see Fig. 1(a)). For 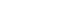, cooperation is promoted. For 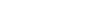, depending on the specific 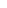 and 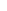, cooperation can be enhanced or suppressed. For 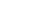, cooperation level plummets to zero, lower than that in the absence of migration, regardless of the values of 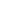 or 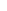. It is shown that 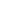 displays discontinuous transitions and the value remains constant between two transition points with increasing 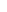. The phase transitions are due to the deterministic “best-take-over” rule we adopt in updating, where a strategy change occurs only if one individual in a C-D pair imitates the other who has a larger payoff (which is also the largest among the neighbors of the former one). Thus phases are altered at the points where C and D have identical payoffs. Considering all equal payoffs of C and D, potential transition points could be: 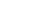, 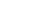, 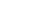, and 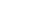, just as shown in Fig. 1(a), where a sharp drop of 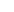 is observed. Altogether, in an environment relatively favorable for cooperators, reputation-based migration enhances cooperation, while in a harsh environment, migration splits the surviving C clusters, accelerating the dying out of cooperators.

The population density 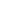 has a notable impact on the dynamical process of evolution [53], since it determines the number of empty sites that serve as destinations of migration, and eventually the characteristics of the network of real interactions [54]. The influence of 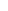 is shown in Fig. 1(b). In the case of no migration, cooperators survive throughout the whole range of 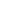, and 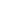 displays a U-shaped curve. By contrast, under our migration mechanism, there exists a threshold of 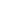 below which 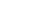. In other words, cooperation can not persist for relatively low population density. However, as 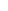 increases, cooperation begins to emerge and flourish. For 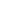 approaching 1, 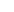 declines sharply to the same value as the no migration case. These phenomena can be intuitively interpreted. For a low density population without mobility, the isolated individuals (also called “frozen sites” that never have the chances to make interactions or strategy changes) and occasionally formed C clusters to a certain extent maintain the cooperation level. Once mobility is introduced, the potential C clusters may be devastated. In our model, migration is based on local information. An individual leaves the dissatisfactory place and moves to a random one. In fact, there is no guarantee that the new place be better than the current one. This is quite unlike the pattern in ref [39] that intentionally searches for the best position globally, which facilitates the getting together of cooperators far between. Consequently, in a sparsely distributed population, migration destroys the positive assortment of cooperators brought by spatial structure, rendering local interactions more like a well-mixed scenario, and cooperators vanish rapidly. This is similar to the results obtained in refs [33], [55] for low population density cases, where although different mobility patterns are adopted, likewise, destinations do not rely on nonlocal information but are actually random. For larger population densities, spatially larger C clusters can be formed initially, and migration helps to expand C clusters, thus enhancing the cooperation. As for 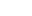, due to the lack of empty sites, migration is almost impossible, and hence the result.

**Figure 2:**
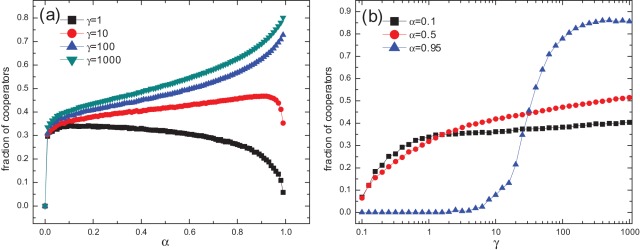
Influences of memory decaying rate and migration sensitivity on cooperation. (a) Fraction of cooperators 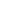 as a function of 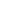 for different values of 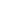 and (b) fraction of cooperators 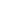 as a function of 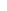 in a logarithmic scale for small, medium, and large values of 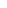. Other parameters are: 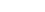 and 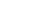. We have excluded the case for 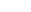 in all simulations because it is unrealistic and it takes infinitely long time for the system to converge as a result of the unlimited growth of reputation value. Other specifications for the simulations are the same as in Fig. 1.

Now we focus on the role of the two important parameters: 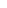 and 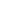, which together govern the motion of individuals. The effect of 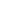 on cooperation is shown in Fig. 2(a). On the one hand, for not very large values of 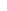 (e.g., 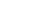 and smaller), neither small nor large value of 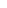 favors cooperation best, but intermediate values of 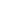 do, although the optimal 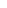 varies with 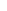. On the other hand, if 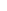 is very large, larger 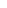 always promotes cooperation. Just as demonstrated in Fig 2(a) for 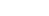 and larger, 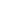 increases monotonously with 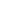. Furthermore, as a common trait for all these 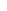
s, a sharp rise in 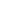 is observed when 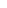 grows slightly larger than zero. The parameter 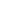, as a decaying factor in reputation scoring, reflects the memory length of the reputation. In our study, we only consider the interval of 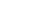 because it is unrealistic to have an infinitely long memory about reputations in society. All the above results indicate that, reputation effect in migration (i.e., a memory length 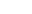) is essential for the establishment of cooperation, but for a limited mobility sensitivity to reputation, memory should not be too long.

**Figure 3:**
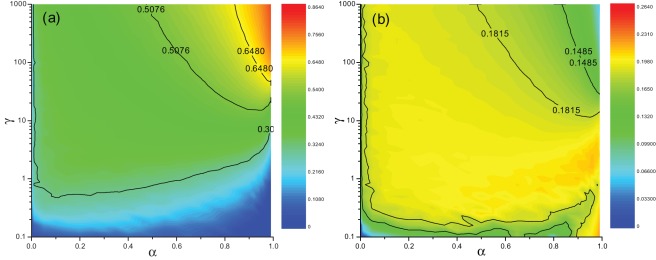
Cooperation levels and migration rates in contour forms. (a) Color-coded values of 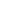 in the 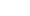 parameter space and (b) corresponding equivalent migration probability. To obtain a data point in (b), the number of migration events is divided by the number of game rounds over a sufficiently long time after the stationary regime is achieved. In both panels, the scale of 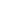 is linear while 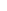 logarithmic, and certain contour lines are labeled. Other parameters are: 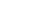, 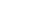. Each data point results from an average over 10 independent realizations.

The influence of 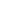 on 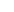 is illustrated in Fig. 2(b). Generally speaking, larger 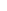
s are better for cooperation for any value of 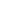. For small and moderate values of 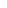, 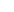 grows monotonously and tardily with increasing 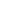. For large values of 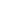 (e.g., 0.95), 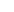 becomes more strongly dependent on 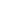 and the curve displays a sigmoid-like shape. A sufficiently large 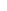 is needed for cooperation to establish, after which 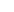 increases dramatically, far exceeding those for lower 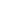
s. The parameter 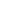 can also be perceived as the capability to distinguish different reputation values. When 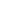 is small, individuals with close reputations are treated alike, and they have similar probabilities to migrate. When 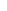 is large, those with higher reputations have larger probability to move but those with lower reputations barely have the chance to move. With larger 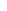
s, individuals' actions are more reasonable or accurate, which favors the evolution of cooperation.

To get a holistic view of the interplay of 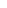 and 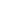, we plot 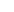 as a function of 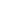 and 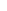 in the contour form (see Fig. 3(a)). It is obvious that for any non-zero value of 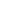, 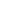 increases with 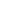. We also find that when 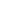 rises, the maximum of 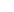 appreciates. Moreover, for relatively small 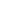
s, the 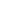 value requisite to sustain the same level of cooperation increases with 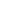, just as demonstrated by the contour line for 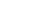. This has led to the non-monotonous behavior of 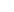 as 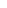 increases for a fixed 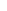, as shown in Fig. 2(a). This phenomenon implies that: For larger values of 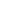, individuals' reputations are established through longer accumulation of previous actions. In these cases, an individual's decision to move or not should be made more accurately, rather than hastily in order to find a better environment for cooperation. However, if 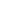 is sufficiently large, to obtain the same 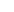 as 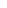 increases, the required 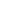 declines(e.g., see the contour line for 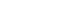 in Fig. 3(a)). The cooperation level is maximized at the top right corner of the plane, which corresponds to 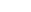 and the largest 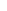. To sum up, we conclude that, larger 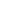 promotes cooperation, as long as the corresponding 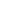 is large enough.

**Figure 4:**
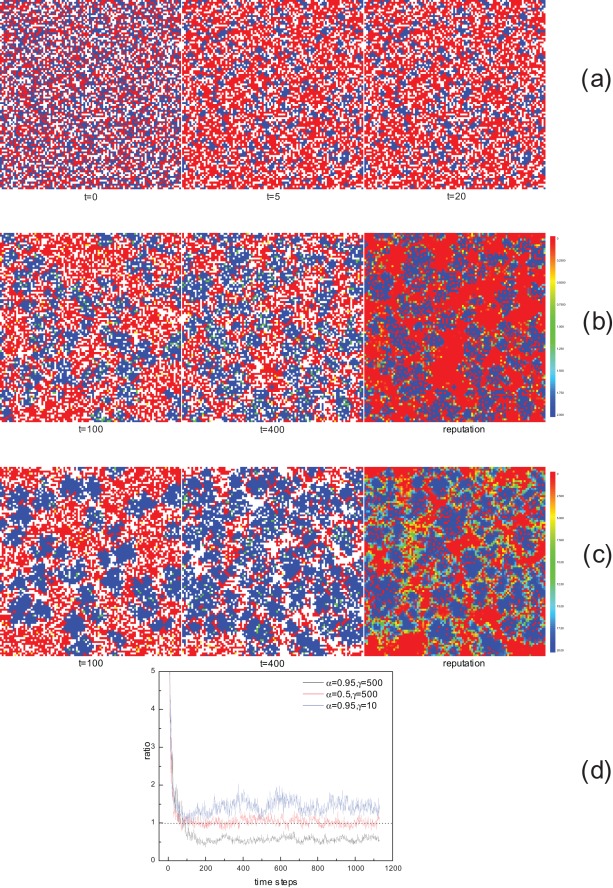
Illustrations of some microscopic properties. (a) Snapshots of distributions of cooperators and defectors at different time steps in the absence of migration. 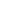 is 0.5, 0.26, and 0.27 for 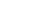, 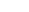, and 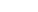, respectively. (b) Snapshots of strategy (left and middle panels) and reputation (right panel, 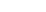) for 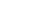 and 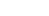. 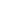 is 0.39 for 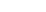 and 0.52 for 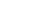. (c) Snapshots of strategy (left and middle panels) and reputation (right panel, 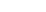) for 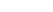 and 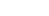. 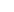 is 0.44 for 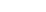 and 0.71 for 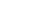. (d) Time evolution of the ratio 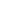 of migration probability of boundary Cs to that of interior Cs. The dotted line 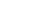 is shown in the figure as a baseline. We have checked that by the time 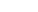 for (a) and 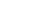 for (b) and (c), the system has reached the equilibrium state. The color coding for strategy snapshots is as follows: blue or green represents a cooperator; red or yellow represents a defector; white represents an empty site. Particularly, green and yellow ones are those who will migrate to new places in this time step. For all the above panels, simulations are carried out for 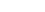, 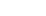, and one realization, starting from the same initial strategy distribution shown in Fig. 4(a) the left panel (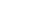).

Reputation induces diversity [20] in migration rates, and the combination of parameters 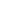 and 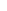 changes the migration probability of individuals as a whole. Figure 3(b) shows the equivalent migration probability as a function of 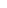 and 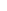 in the contour form after the system has reached the equilibrium. Notably, the migration probability reaches a local minimum at the upper right corner of the plane, which coincides with the highest 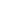 in Fig. 3(a). Roughly, a larger cooperation level corresponds to a lower migration probability, and otherwise leading to the opposite. However, some exceptions appear near the lower boundary of the parameter plane, where both the 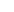 and the migration probability are low. This is due to the constraint in our model that an individual in an all-zero reputation neighborhood (including himself) just stays still. Within this parameter region, population is mainly composed of defectors. Their reputations quickly fall to zero and hence they no longer move, reducing the migration probability.

**Figure 5:**
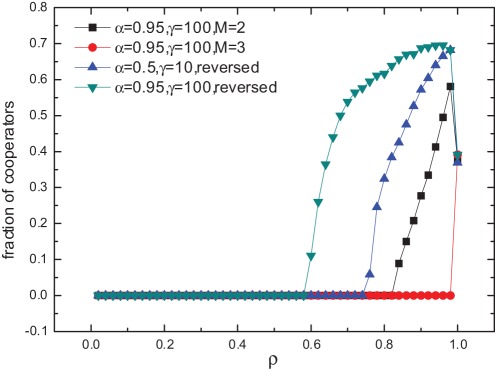
Influences of migration range and the order of migration and imitation. Fraction of cooperators 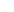 as a function of population density 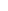 is shown in the figure for 4 different cases. For the first two cases, the migration range is enlarged to the Moore distance of 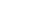 and 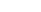, respectively. For the other two cases, the order of migration and imitation is reversed such that migration comes after the strategy imitation. For all the above cases, 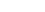.

For better understanding the mechanism of migration based on reputation and the varying effects of different parameters, it is useful to probe into spatio-temporal properties of the evolution process. We first consider the case that migration is not allowed. Figure 4(a) shows the snapshots of spatial configurations of strategies and empty sites at different time steps. Initially, owing to the randomly distributed strategies, those solitary cooperators sparsely scattered among defectors rapidly die out, and only those small C clusters survive (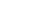). Then these small C clusters expand to the limit, a state both Cs and Ds can resist the invasion of each other on the basis of payoff advantages, and the system quickly reaches a frozen state where no further strategy changes occur (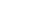). However, if the reputation-based migration is incorporated, the limitation for strategy propagation is broken (see Fig. 4(b), 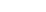), C clusters disperse and occupy larger areas, resulting in a remarkable rise in cooperation level. Meanwhile, C clusters become looser, with many empty sites in them. Eventually, system reaches a dynamic equilibrium (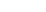), where part of individuals still tend to move. While expanding, some C clusters may split into smaller ones or vanish by chance, thus keeping 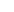 stable on average. If the parameter 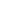 is further increased (see Fig. 4(c)), the C clusters become more compact, with less holes in them. Besides, less individuals tend to move in one step, partially because of the direct influence of parameter itself on migration probability, and partially because of the less empty sites inside the more densely assembled C clusters. Therefore, the real migration rate gets reduced, just as shown in Fig. 2(b). Moreover, there are some filament-like Ds separating or along the edge of C clusters. With almost zero reputation values, these Ds enjoy staying with reputation-high Cs and never move away. This also constitutes a reason that further expansion of Cs is inhibited.

We have also plotted the snapshots of reputations when system is stabilized in Fig. 4(b) and Fig. 4(c) (the right panels). We see that the high-reputation areas coincide with the C clusters. Moreover, when 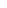 is larger, there will be more individuals with medium values of reputation. Actually, the parameter 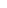 also determines the maximum reputation value. For an individual that always cooperates, his reputation approaches 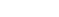, just as illustrated in the snapshots. For a larger 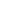, reputations show a more smooth transition from the highest to zero centering on C clusters. This is crucial for stabilizing cooperation, because the hierarchies in reputation values deter individuals in the vicinity of high-reputation ones from moving away, and this “viscosity” spreads by layers, leading to more compact C clusters.

Cooperators at the boundary of C clusters may have different migration probabilities from those in the interior of C clusters. We say a cooperator is at the boundary if he is adjacent to at least one defector, or in the interior otherwise. By 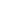 we define the ratio of the average migration probability of boundary Cs to that of interior Cs, and the time evolution of 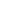 is illustrated in Fig. 4(d). It is found that large 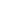 and 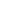 correspond to small 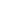. In turn, a decrease in either 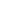 or 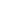 results in a larger 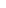. We should stress that a relatively low boundary migration probability is essential in promoting cooperation under our mechanism. If a cooperator at the boundary moves too frequently, it is quite likely that he jumps into a sea of defectors and finally dies out. Nevertheless, a high migration rate makes clusters looser, thus more susceptible to defectors. Therefore, the ideal mode is that: Cs at the boundary hold the position and defense the frontier, waiting for the reinforcement of Cs from the interior. When local advantages are formed, they strike back and expand the territory. In our model, high reputation individuals are more likely to be dissatisfied and tend to move. By contrast, low reputation ones will be pleased to stay next to the high ones. Consequently, to make C clusters stable and compact, on one hand, reputation hierarchy is needed, and on the other, migration can not be made too frequently or blindly [32], [34], [37]. Both of them require a large 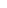 and a large 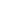.

We also investigate the robustness of the results by reversing the order of migration and imitation (see Fig. 5), and find that the results are qualitatively unchanged. However, the cooperation level is slightly reduced and it requires larger population density for cooperation to establish. The reduction of cooperation should be ascribed to the fact that, if the strategy imitation precedes migration, an individual will not be able to avoid the bad environment in advance [32]. Additionally, we have checked the case in which the migration range is enlarged and find that cooperation is not further promoted (see Fig. 5). The results simply imply that, without a knowledge of the information on destinations of migrations [41], [47], [55], a long range move could be more risky, or sometimes disastrous, because the movement may be from a bad place to an even worse one.

## Discussion

To sum up, we propose a new model to investigate how mobility affects the evolution of cooperation. In this model, individuals evaluate their environments by the reputations of neighbors and themselves. An individual tends to leave his current place and to migrate to a random nearby site if his reputation is higher than those of neighbors. We explore the effects of the two intrinsic parameters: reputation memory length and mobility sensitivity to reputation. It is found that both the memory length and the mobility sensitivity to reputation significantly change the dynamical process of evolution. We conclude that this migration mechanism enhances cooperation for relatively large population density and not very high temptation to defect. For any given reputation memory length, a higher mobility sensitivity to reputation is always better for cooperation. As reputation memory length increases, cooperation can be maximized at a higher level given that the corresponding sensitivity is large enough. We also provide a microscopic perception of the effect of this mechanism.

In agreement with previous studies [32], [33], [37], our results show that high probabilities of mobility inhibit cooperation. The reputation-based migration mechanism also induces diversity in migration probabilities, which have been shown to promote cooperation [37], [38]. Different from theirs, the diversity in mobilities in our model is not a prescribed assumption, but a consequence of the reputation-based migration. Thus it is more reasonable. Our work may shed some light on how migration affects the evolution of cooperation.
